# Top-down control of human motor thalamic neuronal activity during the auditory oddball task

**DOI:** 10.1038/s41531-023-00493-1

**Published:** 2023-03-27

**Authors:** Frhan I. Alanazi, Suneil K. Kalia, Mojgan Hodaie, Adriana L. Lopez Rios, Andrés M. Lozano, Luka Milosevic, William D. Hutchison

**Affiliations:** 1 Department of Physiology, University of Toronto, Toronto, ON Canada; 2 Krembil Brain Institute, Leonard St, Toronto, ON Canada; 3 Department of Basic Sciences, Prince Sultan bin Abdulaziz College for Emergency Medical Services, King Saud University, Riyadh, Kingdom of Saudi Arabia; 4 Division of Neurosurgery, Toronto Western Hospital, 399 Bathurst Street, Toronto, ON Canada; 5 Department of Surgery, University of Toronto, Toronto, ON Canada; 6 Hospital Universitario San Vicente Fundación, Medellin (Rionegro), Colombia; 7 Institute of Biomedical Engineering, University of Toronto, Toronto, ON, Canada

**Keywords:** Neurophysiology, Parkinson's disease, Systems biology

## Abstract

The neurophysiology of selective attention in visual and auditory systems has been studied in animal models but not with single unit recordings in human. Here, we recorded neuronal activity in the ventral intermediate nucleus as well as the ventral oral anterior, and posterior nuclei of the motor thalamus in 25 patients with parkinsonian (*n* = 6) and non-parkinsonian tremors (*n* = 19) prior to insertion of deep brain stimulation electrodes while they performed an auditory oddball task. In this task, patients were requested to attend and count the randomly occurring odd or “deviant” tones, ignore the frequent standard tones and report the number of deviant tones at trial completion. The neuronal firing rate decreased compared to baseline during the oddball task. Inhibition was specific to auditory attention as incorrect counting or wrist flicking to the deviant tones did not produce such inhibition. Local field potential analysis showed beta (13–35 Hz) desynchronization in response to deviant tones. Parkinson’s disease patients off medications had more beta power than the essential tremor group but less neuronal modulation of beta power to the attended tones, suggesting that dopamine modulates thalamic beta oscillations for selective attention. The current study demonstrated that ascending information to the motor thalamus can be suppressed during auditory attending tasks, providing indirect evidence for the searchlight hypothesis in humans. These results taken together implicate the ventral intermediate nucleus in non-motor cognitive functions, which has implications for the brain circuitry for attention and the pathophysiology of Parkinson’s disease.

## Introduction

Selective attention is vital for an organism to survive as the relevant signals and stimuli in the environment need to be filtered out of irrelevant background information. This mechanism was postulated to deal with the “bottleneck” situation since not all the myriad of information can be processed by the brain. Traditionally, the two main bottleneck theories are that of Broadbent^1^ and Treisman^2^ where the Broadbent theory states there is an elimination of irrelevant information and the Treisman theory suggests that this information is not completely lost but strongly attenuated (see Mcleod)^3^. The Treisman attenuation model helped explain the cocktail party effect where despite active filtering out all background conversations to attend to the conversation at hand, the individual can still pick out his/her name from that unattended background. Attentional control can be further divided into bottom-up and top-down mechanisms. While bottom-up attention refers to external sensory signals required for the task at hand, attentional control under internal guidance is well known as a top-down mechanism^4^. This top-down modulation was first shown in the thalamus and gave rise to the searchlight hypothesis. The searchlight hypothesis was formulated initially by Anne Treisman and Francis Crick proposed that the thalamic reticular neuron (TRN) was the site of this regulation or “gatekeeper” and was activated when attention was needed. The searchlight hypothesis proposed a mechanism in which the thalamus controls selective attention by focusing only on one specific feature at any given time. In this model, the thalamic reticular nucleus inhibits the irrelevant thalamic information and allows relevant ascending sensory information to pass up to the cortex^5,6^. TRN is a layer of interneurons that inhibits the thalamic core via GABAergic projections^7^.

Despite being initially described as a “relay station” based on similar neurophysiological receptive field properties of the lateral geniculate nucleus (LGN) to the primary visual cortex, the LGN was then found to change receptive fields depending on the locus of attention. Evidence on the gating roles of the thalamus and TRN was investigated in animal systems. In macaque monkeys, McAlonan et al.^8^ observed a modest decline in activity of TRN neurons adjacent to the LGN before an increase in LGN activity during visual attention^8^. In a mouse model using a cross-modal task requiring selection between visual and auditory signals, LGN in the auditory attending task showed a decline in firing rate while visual TRN showed the opposite effect^9^. Thus, TRN inhibition of the sensory thalamus aids in attending to appropriate sensory information. An additional key player in the process is the prefrontal cortex in determining the rule for attentional control^10^. Nakajima, et al.^11^ discovered that the prefrontal cortex projected to the TRN through the basal ganglia in auditory and visual attention^11^. Thus, it was shown in rodents that the role of TRN in gating the thalamus from higher cortical control was through the basal ganglia. However, since the human motor thalamus has a unique population of inhibitory interneurons receiving cortical inputs^12,13^, it is possible that a faster, more direct route exists.

Most studies of this selective gating have been performed on the sensory thalamus and associated part of TRN in animal models. However, TRN projects to both sensory and motor thalamic nuclei^7,14^. Ventral thalamic nuclei involved in motor functions receive input from the basal ganglia (ventral oral anterior, and posterior nuclei, Voa, Vop, resp.) and cerebellum (ventral intermediate nucleus, Vim). Anatomically, Vim contains the medium-sized neurons that project to the cortex and smaller local, inhibitory interneurons that connect with each other and with the projection neurons. Most of the neurons in Vim respond to limb or joint movement which implicates the Vim as a proprioceptive region^15,16^. Vim receives ascending projections from the spinothalamic tract and vestibular projections and projects to the motor cortex to process motor information^17^. Recent studies have implicated Vim as a gateway for the incoming information required to trigger movements and its thalamocortical projection yield an appropriate motor action. Hence, stimulation of Vim has been shown to relieve the tremor in essential tremor (ET) and tremor-dominant Parkinson’s disease (PD) either by deep brain stimulation (DBS) or by magnetic resonance-guided focused ultrasound^18^.

The Voa/Vop region of the thalamus receives input from the globus pallidus (the output nucleus of the basal ganglia) and this pallido-thalamocortical motor system has been demonstrated to be involved in cognition. The globus pallidus internus (GPi) was shown to have cognitive and attentional roles^19^, and we have previously shown selective responses of GPi neurons as well as beta desynchronization of the local field potentials (LFPs) to the attended tones but not for unattended tones^20^. In an event-related potential study, Vim has been shown to be involved in the early target detection processing of the visual cues of the oddball task^21^. Nevertheless, there is little evidence that demonstrated the control of human Vim and Voa/Vop in selective attention and top-down mechanisms.

Since we found selective excitatory responses in GPi to deviant tones and since GPi sends inhibitory projections to the motor thalamus, we hypothesized that the Vim neuronal activity should be selectively inhibited by the deviant tones in the auditory oddball task. In the current study, we found Vim firing was inhibited when patients selectively attended to the deviant tones but were surprised to find a strong inhibition of firing of motor thalamus neurons during the cognitive task. This is indirect evidence of the TRN gating theory in humans. Analysis of the LFPs further suggested the involvement of Vim beta (13–35 Hz) oscillations in attention and sensory-related cognitive functions.

## Results

A total of 25 tremor patients undergoing microelectrode recording prior to Vim DBS were included in the study. Nineteen patients were included in the LFPs analysis, the remainder had closed filter settings (cut below 300 Hz) to control higher noise in those recordings. A total of 42 trial sequences or blocks of 50 trials of an auditory oddball task were conducted with randomly occurring infrequent deviant tones. Most patients reported the correct number of deviant tones (*n* = 19, 76%) while six patients (24%) reported an incorrect number of tones. Tasks were classified as correct if the count was ±1 to the actual number of deviant tones, otherwise, it was classified as incorrect.

### Spiking activity

A total of 56 neurons was recorded from the motor thalamus in 42 blocks of 50 trials (single tone presentation) in 25 tremor patients. When these neurons were localized to the subregion of the motor thalamus, 38 neurons (68%) were found in the Vim, and 18 neurons (32%) were localized more anteriorly in the Voa/Vop area. The baseline firing rate for both nuclei before the task was 25.9 ± 2.3 Hz. The average firing rate of motor thalamic neurons over the task block was 18.7 ± 2 Hz, significantly lower than the baseline firing rate *(F* = 11.04, *p*-value = 0.002). The firing rates of the neurons after the task block were over-rebounded to 22.3 ± 1.9 Hz *(F* = 0.425, *p*-value = 0.519). Vim baseline firing rate was 28.4 ± 2.7 Hz where Voa/Vop mean firing rate was 17.8 ± 3.3 Hz*)*. Vim firing rate during the task was 19.9 ± 2.2 Hz *(F* = 4.57, *p*-value = 0.0418) and after the task was 22.9 ± 1.8 Hz *(F* = 0.319, *p*-value = 0.577*)*. For Voa/Vop, the firing rate over the task was 14.9 ± 4.5 Hz *(F* = 33.9, *p*-value = 0.002*)*. and after the task was 20.3 ± 5.9 Hz *(F* = 0.574, *p*-value = 0.483*)*. The averages of firing rates in the motor thalamus before, during, and after the task are shown in Supplementary Fig. 1. We also tested an additional five neurons in the putative thalamic reticular nucleus with bursting activity at the start of trajectories targeting the subthalamic nucleus in a separate study, but none show a significant modulation in firing rate or pattern.

**Fig. 1:**
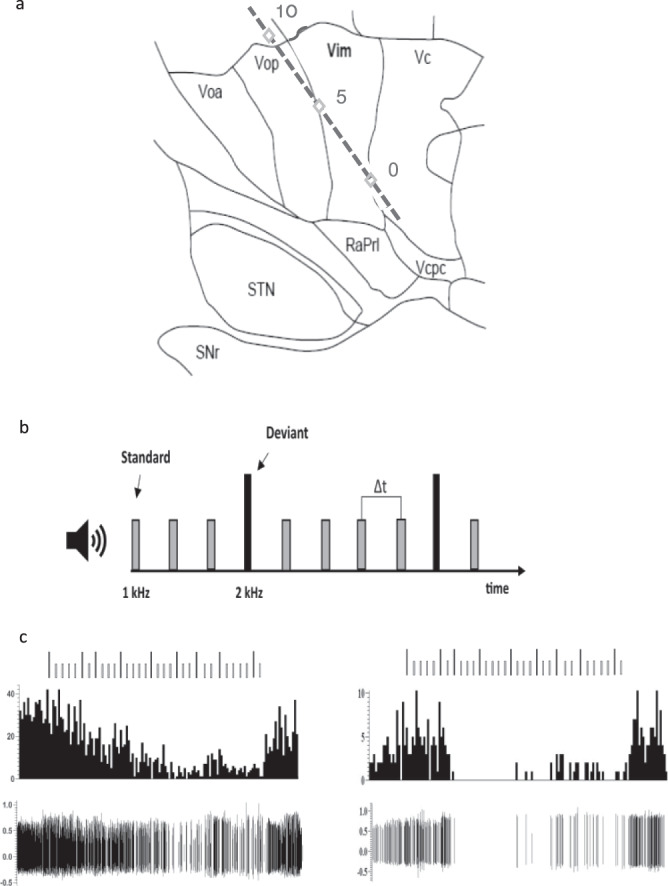
A typical microelectrode track and the behavioral task. **a** A representation of the microelectrode track through the motor thalamus targeting the Vim/Vc (0) border passing through Vop. A second series of trajectories (not shown) were more anterior and targeted the Vop/Vim border and passed through Voa. Track is presented on a 14.5 mm sagittal map from Schalterbrand and Wahren^47^. A few recordings were made from thalamic reticular nucleus in tracks targeting the STN located more anterior still. RaPrl prelemniscal radiations, Vcpc ventral caudal parvocellular. **b** A schematic representation of the auditory oddball task. Patients were presented two different tones standard (1 KHz, 80% of the tones) and deviant (2 KHz, 20% of the tones) with the same interstimulus time. Patients were asked to count and report the number of deviant tones. In five trials, patients were also asked to move their wrist during the deviant tones. **c** Examples of two Vim responses to the behavioral task. Vim neuron showed a striking inhibition during the attentional task characterized by a gradual attenuation of the firing (*left*) or a complete shutdown of the neurons (*right*).

We also analyzed whole block firing separately for the group of 44 cells recorded during the correct count block of trials. The baseline firing rate was 25.2 ± 3.7 Hz and the firing rate during attending to the cognitive task was 17.4 ± 3.56 Hz, significantly lower than that of the baseline *(F* = 8.94, *p*-value = 0.006). Figure 1 lower panel shows two such neurons in the motor thalamus that were strongly (or at times completely) inhibited during the correct performance of the cognitive task. The inhibition was not immediate at the start of the task but showed a slow attenuation as the task progressed (right) or an abrupt cessation of activity with some breakthrough firing (left), In both cases the firing returned to baseline around the end of the trial block and reporting of the count. Twelve neurons were recorded during incorrect count reports. The firing rate at baseline was 22.6 ± 5.6 Hz and during the task was 20.0 ± 4.6 Hz. The firing rate during the tasks with incorrect count reports did not differ significantly compared to baseline *(F* = 1.914, *p*-value 0.239), indicating that attention and\or counting during the task was causing the inhibition. The normalized firing rates changes to the correct and incorrect tasks are shown in Supplementary Fig. 2. Pre-trial sequence firing pattern was compared to the pattern during the trial sequence. In the PD group, 11 neurons had a decreased burst index during the task whereas four increased, and one did not change by more than 0.5. In the ET group 18 neurons had a lower burst index during the task, three increased and three cells did not change.

**Fig. 2:**
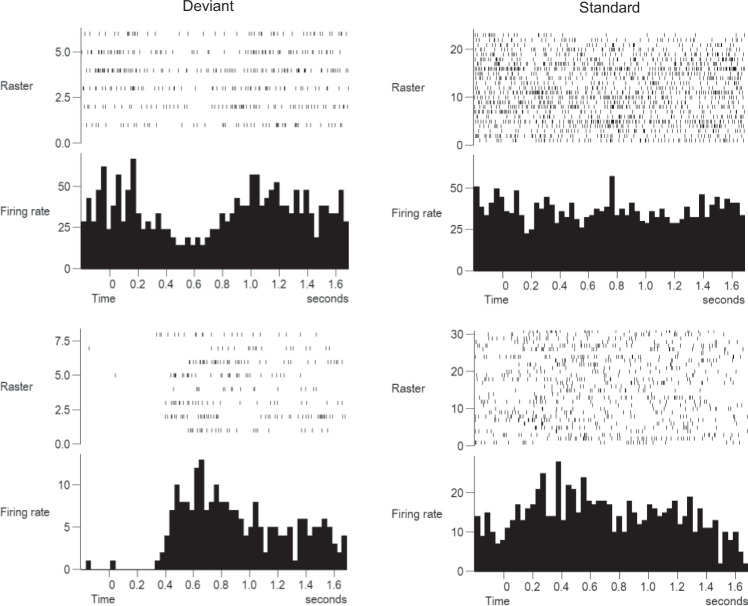
Peristimulus time histogram of responses to the attended and unattended tones. An example of different responses to the auditory oddball task. In most neurons, there was a decrease in the firing rates during the deviant tones whereas no change was seen to the standard tones (*top-panel*). Some neurons showed an increase in firing rates (*bottom panel*). This increase was selective to the deviant tones that patients were asked to count.

**Fig. 3:**
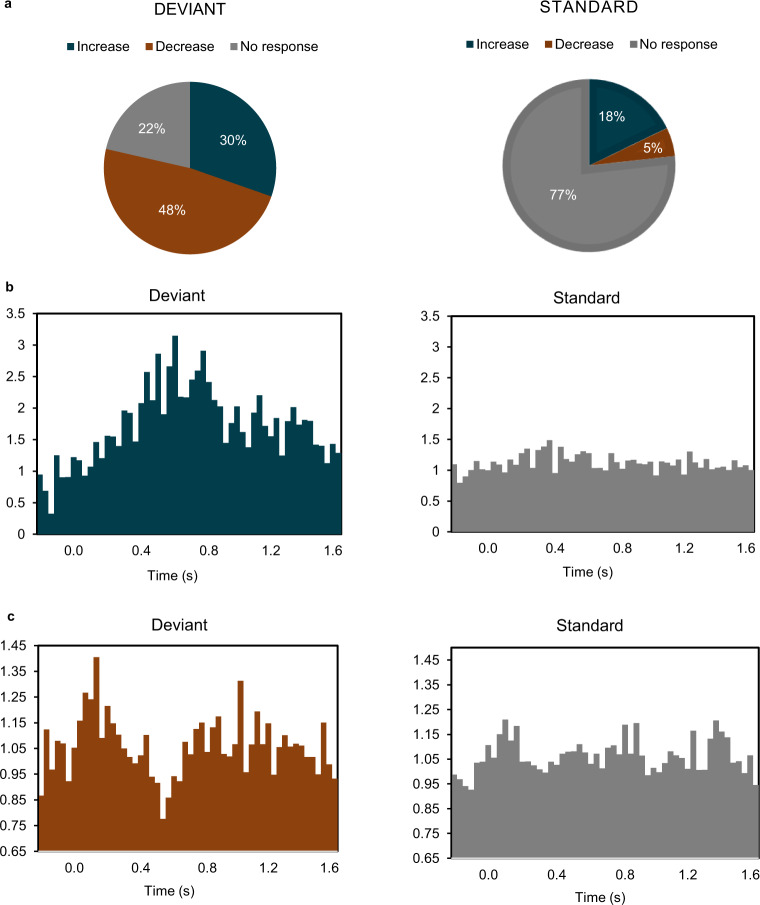
Population average histograms for different neuronal responses to the task. **a** Pie charts show the motor thalamic neurons response to the auditory oddball task. The majority of the neurons (78%) responded to the deviant tones (*left*) whereas only 23% of the neurons showed a response to the standard tones (*right*). The population histograms are averages of the individual cell histograms that showed an increase (**b**) to the deviant tones (left) compared to the standard tones (*right*), and the neurons that showed a decrease in firing rate (**c**).

**Fig. 4:**
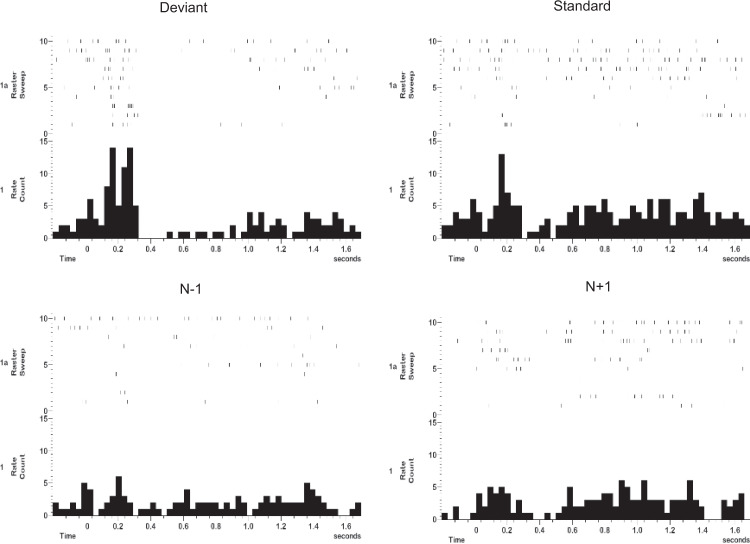
Variable phasic cue responses to standard and deviant tones of the auditory oddball task. A phasic as well as tonic response was observed in some thalamic neurons. In this example, a phasic response is present in response to the deviant (“N”) (*upper left*) and in the first and last five standard tones (*upper right*). A tonic inhibitory response was more prominent for the deviant tone. Histograms in lower panel show averages of all the standard tones just before (*N* − 1) and just after (*N* + 1) the deviant tone and no phasic response was prominent.

Comparing the within-trial responses between the two types of tone, 30 neurons (78.9%) in Vim showed a selective response to the deviant tones, whereas eight cells (22.1%) showed no response to the deviant tones. Fourteen of 38 cells in the Vim showed a decrease in the firing rate and 16/38 neurons increased during the deviant tones. Examples of the selective decrease and increase of motor thalamus neurons to the deviant tones are shown in Fig. 2. In the top left panel, there appeared to be more bursting evident in the raster plot and histogram before the tone was presented and less bursting during the inhibitory response to the deviant tone. This was not apparent for standard tones. Most of the cells (*n* = 29, 76.3%) did not respond to the standard tones, and nine neurons showed either an increase or decrease in the firing rates (see Fig. 3 and Supplementary Table 1). Voa/Vop cells showed similar selective responses to deviant tones as 14 out of 18 cells exhibited an increase or decrease in spiking activity and four showed no change in firing rates. Three neurons were excited, and 11 cells were inhibited during the deviant tones. An early phasic cue response to the deviant tones was found in 15 neurons, whereas this response was present in three neurons following the standard tones. An example of the phasic cue response with a decrease afterwards for the deviant and standard tones is shown in Fig. 4. In this example, the cell showed a decrease for both tones after the cue response with more inhibition to the deviant tones than the standard tones.

### Local field potentials

**Fig. 5:**
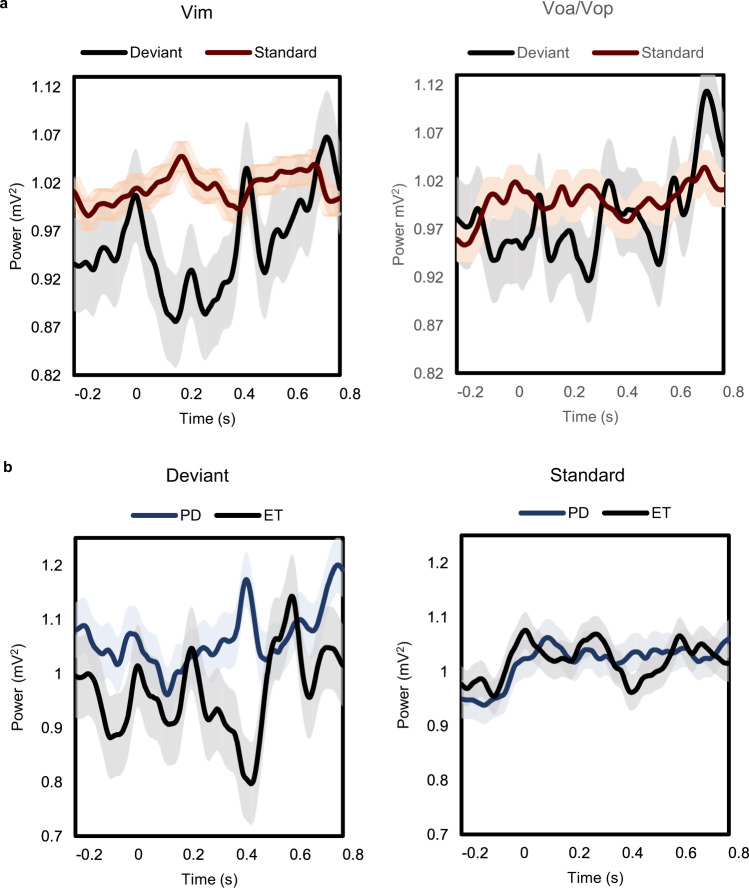
Averages of beta local field potentials in the motor thalamus and different disease states. **a** Total averages of beta band (12–35 Hz) LFPs in the Vim (*left*) and Voa/Vop (*right*) for the deviant (red) and standard tones (blue). Beta LFPs modulations were observed to the attended tones in the Vim and Voa/Vop. However, a clear modulation is seen in Vim in the cognitive window (0.2–0.45 s) to the deviant tones where patients asked to count and not for the standard tones where patients asked to ignore. **b** beta LFPs divided by the disease type. LFPs in the beta band in response to the deviant tones (left) and the standard tones (right). Blue line is Parkinson’s disease tremor (*n* = 4), and black line is essential tremor (*n* = 15). Clear beta modulations (desynchronizations) are observed in the motor thalamus of ET group but not in the PD patients. These comparisons were significant in The Mann–Whitney U test. Results are mean ± SD.

Neuronal oscillatory activity in the beta band frequency (13–35 Hz) was recorded in the Vim and Voa/Vop while tremor patients performed the auditory oddball task. Significant differences in the power of beta LFPs were found in the different subnuclei of the ventral thalamus. The overall baseline average amplitude (power) of beta activity LFPs in all patients was 0.116 ± 0.0019 mV^2^. The baseline mean average beta activity in the Vim was 0.105 ± 0.0023 mV^2^, and in the Voa/Vop was 0.128 ± 0.0021 mV^2^ *(t* = −213.0, *p*-value < 0.001). In response to the task, the deviant tones were followed by a phasic beta burst response with beta band desynchronizations during the period between 0.25 s to 0.6 s (Mean = 0.969 ± 0.0286 mV^2^), whereas the standard tones did not show the same modulation (Mean = 1.013 ± 0.0153 mV^2^, see Fig. 5a) *(t* = −45.0, *p*-value < 0.001). For the statistical analysis, we also compared the pre-stimulus time to the post-stimulus time frames. This revealed a statistical significance in beta LFPs power between deviant and standard tones in the times between 0.05 to 0.2 s (auditory sensory epoch, *t* = −2.256, *df* = 23.44, *p*-value = 0.0337) and 0.21 to 0.6 s (“cognitive window” epoch, *t* = −2.545, *df* = 21.40, *p*-value = 0.0187). There was no significant difference in the pre-stimulus time epoch between −0.2 s to 0 (*t* = −1.176, *df* = 23.65, *p*-value = 0.2513) and >0.6 s (recovery epoch, *t* = −0.677, *df* = 26.11, *p*-value = 0.5045). In Vim, a clear beta desynchronization was seen following the deviant tones. There were further beta modulations after the tone onset with two beta peaks or bursts at 0.45 s and 0.75 s. Voa/Vop region showed less modulation to the deviant tones than the Vim. Voa/Vop beta modulation starts before the deviant tones and elicited a phasic burst at 0.1 s with the other two bursts at 0.35 s and 0.75 s. The overall averages of beta oscillatory activity in the Vim (left) and Voa/Vop (right) is shown in Fig. 5a. We further divided the patients based on the disease states to compare PD with ET. PD tremor patients had more beta than ET patients and orthostatic tremor patients. The overall baseline average activity for PD tremor (*n* = six) was 0.2648 ± 0.0174 mV^2^. In comparison ET patients, the overall baseline average of beta LFPs was 0.0795 ± 0.0044 mV^2^ *(t* = −327.45, *p*-value < 0.001) and for the orthostatic tremor patients was 0.0685 ± 0.0043 mV^2^. For the behavioral task, ET patients showed a clear beta modulation (desynchronizations) for the deviant tones but not for the standard tones (Deviant = 0.9636 ± 0.0754 mV^2^, Standard = 1.0195 ± 0.0327 mV^2^) *(t* = −53.64, *p*-value < 0.001), where PD tremor patients did not show a clear modulation although it was statistically significant (Deviant = 1.0628 ± 0.0505 mV^2^, Standard = 1.0169 ± 0.0341 mV^2^) *(t* = 20.97, *p*-value < 0.001). The overall averages of the PD tremor (*n* = 6) and ET (*n* = 15) in response to the deviant (left) and standard (right) tones are shown in Fig. 5b.

### Cognitive and motor tasks

**Fig. 6:**
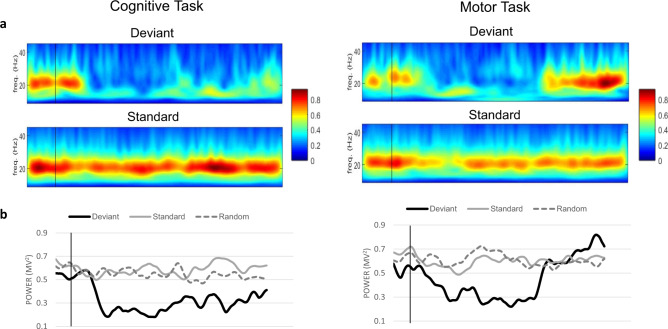
Neuronal responses to the cognitive and motor tasks. **a** Event related spectral analysis (ERSA) of LFPs from open filter and smoothed microelectrode recordings in response to the cognitive task (Left) and the motor task (rapid wrist extension, Right). The ERSA is showing an individual example of the LFPs modulations where beta LFPs activity aligned to the onset of tones (black line). Top spectrogram is the beta LFPs response to the deviant tones and the bottom spectrogram is showing the response for the standard tones. The post-stimulus time frame is 1.8 s. Beta LFPs activity is showing a beta desynchronization around 0.2 s for both tasks but more prolonged desynchronization for the counting only task (cognitive task). **b** Line representations of the same examples for both tasks comparing beta LFPs activity for the deviant tones with standard tones and with random triggers.

**Fig. 7:**
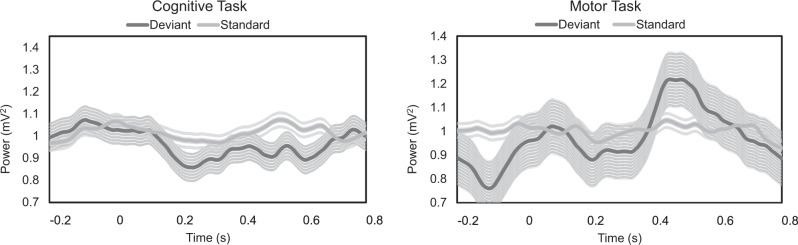
Averages of beta LFPs for the motor and cognitive tasks. Averages of LFPs in the beta band frequency for the cognitive (*left*) and wrist flicks task (*right*) (*n* = 5 sequence of trials). Cognitive task reveals a beta desynchronization after the tone around 0.2 s and the rebounds around 0.8 s. On the same neurons, wrist flicks are showing a similar pattern of beta desynch but with beta burst around 0.6 s. Results are mean ± SD.

In another trial sequence, patients were asked to first perform the auditory oddball task, silently count the tones, and then the same units were tested again in another trial sequence where patients extended the wrist (“wrist-flick”) to every deviant tone in the trial. The total trial sequences were 5 trials for both tasks. In the silent counting trial sequence, the firing frequency at baseline decreased from 28.4 ± 5.2 Hz to 19.6 ± 7.0 Hz during the task (*P* < 0.05). For the wrist-flick task, the average reaction time from the onset of the tone to the onset of wrist flick was 0.311 ± 0.015 s. The firing rate at baseline was 28.4 ± 6.1 Hz. During the motor task, the firing frequency increased to 36.1 ± 7.3 Hz, a rate significantly higher than the baseline (paired t-test, two-tailed, *P* < 0.05). Comparing the two tasks, the mean firing frequency was significantly higher in the wrist flicking task compared to the silent counting task (paired t-test, two-tailed, *P* < 0.01). For beta LFPs, the averages for the cognitive task were (Deviant = 0.966 ± 0.0599 mV^2^, Standard = 1.0148 ± 0.0309 mV^2^) and for the motor task were (Deviant = 0.975 ± 0.114 mV^2^, Standard = 1.004 ± 0.023 mV^2^, Movement = 0.992 ± 0.0973 mV^2^). Both tasks showed a beta desynchronization in the LFPs after 0.2 s with a rebound burst afterwards for the motor task. An example of the desynchronization in the beta LFPs for the cognitive and motor tasks is shown in Fig. 6. The overall averages of both tasks are shown in Fig. 7.

## Discussion

In this study, we investigated neuronal firing rates and LFPs oscillations while implementing an attentional task in the motor thalamus of movement disorders patients prior to DBS implant surgery. Single units and beta LFPs were derived from the same microelectrodes so that a comparison of these disparate signals could be made simultaneously. We observed a near to complete shutdown of the motor thalamus neurons during the cognitive task and the selective modulation of thalamic beta activity to attended stimuli in dopamine intact but not in dopamine-deprived PD patients. Although selective modulation of visual versus auditory cues has been demonstrated in animal models, we show an attenuation of proprioceptive or sensorimotor information by higher order cortical function. This inhibition was specific to patients who attended to the auditory task at hand, as patients who counted incorrectly or did a motor task to deviant tones did not show the same inhibition. We also recorded the oscillatory fluctuations of the LFPs from the Vim and Voa/Vop and showed selective modulations of beta frequency band oscillations in the LFPs of both nuclei to the deviant tones. This neurophysiological evidence supports the involvement of Vim and Voa/Vop of the thalamus in non-motor attentional functions, and extends our previous findings in GPi where we found selective modulation of the beta frequency LFPs and high-frequency discharge neurons during the same cognitive task^20^. However, we assume the control is from higher cortical areas and not pallidofugal since we never saw a progressive increase of GPi firing over the duration of the task trials that would explain the strong attenuation of firing seen in the motor thalamus in the present work.

Beta activity (13–35 Hz) is well known to be involved in the context of motor processing. Beta oscillatory activity was previously found to be desynchronized in the Vim during motor activity^22^. Indeed, desynchronization observed in the current study was associated with selective attending and silent counting alone, ruling out the notion that the beta desynch was due to a motor response. In healthy individuals, cortical occipital beta desynchronization was observed in selective attention to visual stimuli while ignoring somatosensory stimuli^23^. Therefore, beta desynchronization in the Vim appears involved directly or as a consequence of neuronal activity related to selective attention. It is proposed that beta is involved in the top-down control where the content of the signal maintains the current cognitive set, or “status quo” and represents the content-specific re-activation of the endogenous information^24^. However, analysis of the firing pattern revealed more decreased burst index during the task in both groups than before and this is not consistent with the increased rhythmic beta activity that would show increased values of the burst index. In support of this, direct analysis of beta power in the spike train of the attenuated neuron showed a slight decrease in power rather than an increase. Further evidence for a cognitive role of beta in the motor thalamus is the fact that beta desynchronization in Voa/Vop was found before tone onset. This behavior of beta to the deviant tones is similar to that found previously by our group in GPi^20^. This confirms and extends our previous findings since Voa/Vop is considered as a pallidal receiving region whereas Vim is more of a cerebellar receiving nucleus, although this division is not mutually exclusive and there is overlap (see Albaugh and Smith)^12^. The beta modulation is maybe related to the expectation effect as cortical recording showing that desynchronization in the beta power occurred in the auditory and motor cortex when participants predicted or expected the upcoming tone^25,26^. Hence, beta activity in Vim and Voa/Vop may take part in the attentional control circuit involving basal ganglia, cerebellum, and the cortex in sensory information prediction.

A possible consequence of the beta desynchronization in the motor thalamus, which is selective to the deviant tones, is to give rise to the illusion of expanded time that has been reported following deviant tones (but not standard tones). In our study, it was noted that there was a prolongation of time after the deviant tones and before the onset of the next tone and we investigated whether this was a technical issue. However, this illusion has already been described in the literature of the oddball task and it is much more robust with auditory than visual stimuli^27^. Several studies have shown that DBS of the subthalamic nucleus decreases beta oscillations both during and for some minutes after stimulation^28^. In a MEG study that investigated the cortex, it has been suggested that beta oscillations may be involved in time estimation, and they showed that lower beta oscillations were associated with time expansion^29^, consistent with our results. Therefore, we propose that the desynchronization of beta in the thalamus following the deviant tone but not the standard tone is likely responsible for the illusion of prolonged time. Indeed, ongoing beta oscillation appears necessary for the perception of the passage of time.

PD tremor and ET tremor have different etiologies involving basal ganglia vs cerebellar circuits although these circuits converge in the ventrolateral thalamus. Beta oscillations have been found in a small group of PD tremor dominant and ET patients^22^. Indeed, we compared a larger group of PD and ET patients and found that PD patients had more beta power than ET patients. This is overall consistent with the so-called oscillation model of PD that posits an overall increase in beta oscillations in the basal ganglia^30^. It is therefore likely the increased beta derives from the basal ganglia output to the motor thalamus. Furthermore, the selective modulations of beta during the attended tones revealed more modulation in the ET group to the deviant tones, and PD patients did not show the same strong modulations. This is in line with previous reports implicating dopamine in the top-down control since ET patients are not dopamine deficient as were the PD tremor patients who were all 12 h off medication^31^. Parkinson’s patients are well known to have attentional deficits and a recent study investigated whether this deficit was in part related to a lack of dopamine acting in the reward system^32^. However, they did not find a difference between high and low reward incentives for the stimuli to be attended in the PD patients. Our preliminary results suggest that if the attentional deficit arises due to a lack of thalamic beta modulation, a striatal dopamine reward system may not be expected.

Single unit’s histograms showed different responses to the task. In the histograms shown in Fig. 2, the upper histograms showed an inverted U-shaped inhibitory period specific to the deviant tone, but, during the period up to the cue, appeared to have synchronized bursts around the alpha frequency. The lower histogram shows excitatory activity during the deviant tone trials in the period of 200–600 ms (“cognitive window”) with a silent background. Since these appear to be the mirror image of the responses shown in the other histograms, we suggest these may be local inhibitory interneurons in the ventral thalamus. These have been described by Yoland Smith’s group and the interneurons receive contacts from cortical areas as well as contacts from subcortical areas, which is the cerebellum in the case of Vim^12^. These neurons are in an ideal position to gate the information going from the thalamus to the cortex and may be a substrate for the responses observed here, rather than the indirect basal ganglia route proposed in the paper of Nakajima, Schmitt and Halassa^11^. These authors proposed a link from dorsolateral prefrontal cortex to globus pallidus to TRN and then to ventrolateral thalamus or motor thalamus in the mouse. Rodents do not have this population of interneurons in the ventral thalamus and indeed the rodent basal ganglia anatomy differs significantly from the primate. Another interpretation is inhibitory surround mechanism where there are adjacent neurons that response in opposite directions to the stimulus in order to increase response contrast, or focus. However, this is a phasic signal and, in this example, does not tonically increase over the trial as would be expected if it were driving the slow decrease in spontaneous ongoing firing seen in some thalamic neurons during the trial.

In the histograms shown in Fig. 4, the raster of all the standard trials shows some variability in responses. In particular, the early response appeared to be a sensory-driven signal, but when averages were taken aligned to the standard tone just before (*n* − 1) and just after (*n* + 1), there was no response to these standards. The response was mainly attentional because it was prominent for the salient deviant tone but also present for standard tones at the start and end of the trial. The initial responses may be novelty to the start of the task, and then the ones at the end may have been due to the expectation of trial ending. These motor thalamic responses are similar to those described by Kurata^33^, where he cued monkeys to make a movement using three different somatosensory modalities, visual, auditory and vibrotactile, and showed similar phasic responses to all three types of cues^33^. However, he did not use a go/no-go paradigm to show an absence of a cue response when the instruction was not to move. These responses may not be just a sensory response to the auditory cue, but what we chose to call a phasic cue response. It is cueing the system about a salient event not just registering a response. These types of responses underscore the concept of the “cognitive thalamus” processing salient cues at the subcortical level.

We also found at the end of the trial a rebound in spontaneous firing that continued up until the report of the number. This increased activity may be related to the delay period activity characteristic of working memory originally described in the cortex by Fuster^34^ but also seen in similar trajectories through the human motor thalamus^35^. If the delay period activity holding working memory in the cortex is a sustained activity of dorsolateral prefrontal cortex neurons projecting to inhibitory interneurons of the ventral cerebellar motor thalamus, then a sustained inhibition would be the thalamic correlated of delay period activity that ends when the count report is given. In this scenario, the increase in activity after the trial is not due to the end of the trial but rather the report of the working memory. This could be easily tested by delaying the request for the report for a variable interval after the trial ends.

Understanding the control of the motor thalamus by cognitive tasks sheds light on possible mechanisms in symptoms observed in PD patients. It is well known that rest tremor can be provoked by asking the patient to count backwards from 100, and we similarly observed cases of tremor onset after starting the oddball task. We hypothesize that the decrease in sensorimotor input to the thalamus with the cognitive task changes the gain of the central thalamocortical loop which is able to start oscillating, and then couples to the peripheral spinal reflex arc (see Prochazka^36^) but the precise mechanism remains unknown^37,38^. The current study showed also beta desynchronization during the cognitive task of silently counting. Thalamic beta activity was previously found to be inversely correlated with tremor power^22^. Hence, the cognitive task could cause tremor activity through Vim beta desynchronization. Further, freezing of gait is a common symptom observed in PD and is triggered by altered attention, dual tasking and auditory networks^39,40^. In addition, rhythmic auditory stimulation during gait training was found to facilitate attention in healthy and PD populations^41^. Thus, the proposed interaction of sensorimotor and auditory pathways could be further investigated in the pathophysiology and treatment of freezing of gait. Lastly, 30–60% of bilateral thalamotomies on treating tremor-predominant PD or ET showed side effects with cognitive deficits^42^. Our study provided evidence for Vim’s involvement in the attentional task, supporting the finding that bilateral lesions of Vim may cause negative cognitive outcomes to the patients.

There were several limitations in this study. First, there were only five neurons tested in the thalamic reticular nucleus and further work needs to be done here. Nakajima et al. demonstrated a regional-specific TRN project to a specific area in the thalamus^11^. TRN was also subdivided into different types specializing in distinct functions^43^. It is also not possible to test various parts of the large TRN given specific trajectories to surgical targets and these parts may not be at the interface of the auditory and motor sections. Therefore, further study is needed to localize the specific region and cell types in the TRN to gain evidence for its control of the circuit as opposed to local interneurons in Vim. Also, measurement of the medial geniculate nucleus (MGN), the auditory thalamus, was missing in the current study task design. Based on current and animal studies, the MGN should have increased firing during the silent counting task and decreased during the wrist-extension task^11,44^. Such inhibition of MGN by TRN was also suggested in human neuronal modeling^45^. Our study only had patients with surgical indications to relieve tremor symptoms, thus lacking the generalizability to the healthy population. Healthy animal models can be utilized with a cross-modal motor and auditory task to study the current selective attention mechanism. Lastly, this study included patients with different disease pathologies (cerebellar vs basal ganglia) which may have impacted the difference in beta modulation rather than dopamine status. The results here encourage further studies with a wider clinical and cognitive comparison between these different disease states.

Our study showed Vim and Voa/Vop neurons under strong inhibitory control during an auditory oddball task, suggesting a mechanism in the motor thalamus for gating ascending information to the cortex. Our results give support to the searchlight hypothesis, although we did not directly implicate the TRN in the control of the spotlight but rather suggest local interneurons may be capable of attenuating irrelevant parts of the thalamus during selective attention. We conclude that beta desynchronization in the motor thalamus mediates selective attention. These effects together suggest a more complex role of the “cognitive thalamus” than purely relaying motor information to the cortex. Understanding the mechanism of selective attention in auditory and motor processing can provide insights into movement disorders and may impact therapy in the clinical population.

## Methods

### Patients and behavioral task

A total of 25 patients undergoing DBS for tremor participated in the study. There were fifteen patients who had essential tremor, six patients had Parkinson’s disease tremor, two had orthostatic tremor and one had multiple sclerosis tremor, and one had a dystonic tremor. Out of the 25 patients, eighteen patients were males and seven were females and the mean age for the participants was 71 years old. All patients went to a unilateral DBS to treat tremor. In terms of laterality, sixteen patients had the DBS for tremor on the left side and nine patients on the right hemispheres. The study conformed to the guidelines set by the Tri-Council Policy on Ethical Conduct for Research Involving Humans and was approved by the University Health Network Research Ethics Board. Furthermore, all the patients in this study provided written, informed consent prior to taking part in the study.

Patients performed a two-tone auditory oddball paradigm with an inter-tone interval of 2 s. Standard-low tones (1000 Hz, *p* = 0.8) and deviant-high tones (2000 Hz, *p* = 0.2) were presented randomly. In each block of trials, there was a total of 50 tones of which 10 were oddballs or deviants. The tone duration was 100 ms. Patients were instructed to silently count the number of deviant tones and report the number at the end of the trial. In addition to the cognitive task, a motor task design was also used in five patients where patients extended their hand about the wrist “wrist flick” to the deviant tones.

### Microelectrode recording and data acquisition

We used dual microelectrode recordings with a spacing of 600 um apart, each extruded from a separate guide tube. This was inserted into the Leksell stereotactic obturator that was used for the insertion of the DBS electrode. In this way, the guide tube was not moved after the microelectrode recordings results were obtained and the DBS implanted. Two surgical approaches were used. One targeting the border of the cerebellar motor thalamus (Vim) and the somatosensory ventral caudal (Vc) and implanting 2 or 3 mm in front of this without recording a further trajectory. The other was targeting more anteriorly the basal ganglia receiving motor thalamus at the Vop border with the Vim and implanting the DBS electrode in that territory. Due to these different surgical approaches, a larger area of the motor thalamus was sampled in the current study. Two microelectrodes (25 um tip lengths, 0.2–0.4 MΩ impedances, sampled at 12.5 kHz) were placed in the Vim and Voa/Vop for recording single unit (cell) activity intraoperatively when patients underwent DBS (Fig. 1). The recordings were low-pass or open filtered (5–3000 Hz) and amplified 5000× using two Guideline System GS3000 amplifiers (Axon Instruments, Union City, USA), Recordings were then monitored and saved through Spike2 software (Cambridge Electronic Design). Techniques for intraoperative identification of Vim have been previously published^16,46^. Briefly, MRI imaging identified the stereotactic coordinates of the anterior commissure and posterior commissure, with the 14.5 mm sagittal section of the Schaltenbrand and Wahren (1977) standard atlas^47^. Vim was localized and then the two microelectrodes were advanced through the calculated coordinates and trajectory.

### Track reconstruction

In order to assign neurons to the Vim or Voa, Vop group, we reconstructed the locations of neurons by adjusting the initial planned trajectory based on the physiological findings. This included the proximity to Vc or the prelemniscal radiation (R.a.p.r.l) based on location but also on the locations where microstimulation (100–200 Hz, 100 µA, 2–5 s, 0.3 ms pulse width) induced paresthesia sensations. Microstimulation in Vc produced tingling sensations localized to an individual finger or part of the hand (the “projected” field) at low stimulation thresholds, (2–10 uA) often with a tactile receptive field at the same or nearby site. R.a.p.r.l. was identified by sites where microstimulation produced a large hemi body field or combined upper and lower limb tingling sensations at thresholds above 20 uA. The Vim/Vop border was estimated by the auditory identification of beta activity in the spike trains of neurons, which often had a higher firing rate compared to neurons more anterior to this border, whereas kinesthetic neurons could be found in both regions.

### Offline neuronal analysis and statistics

Data were analyzed offline using Spike 2 (Cambridge Electronic Design) to measure the single neurons and local field potentials. To study the single unit responses, the raw signal was filtered through a finite impulse response filter, bandpass second order of 300–3000 Hz, to separate spikes from background activity. Action potentials and associated units were isolated, and a spike train was generated using a template matching tool in Spike2 to measure neuronal spike trains. Event channel triggers were generated by detecting the peak frequency of deviant and standard tones. For wrist-flicking tasks, reaction time, the time from deviant tone to the onset of movement recorded from the electromyography recording, was calculated. Neuronal responses and LFPs were further analyzed through MATLAB with the in-house gaussian inter-stimulus interval and event-related spectral analysis scripts. Single unit activity was analyzed utilizing peri-stimulus time histograms for the deviant and standard tones. Peri-stimulus time histograms were considered to have a significant response if three consecutive bins (20 ms) were under or over the one standard deviation of the baseline cell firing^48^. The firing pattern before and during the trial block was analyzed by means of a burst index calculated as the ratio of the mean inter-spike interval divided by the modal inter-spike interval, and a difference of more than 0.5 was considered significant. To analyze the LFPs, Event-related spectral analysis plots (0.2 s offset, 1.6 s onset, 0.20 ms bin size, Morlet parameter 4) aligned to tone presentation were created. Analysis was restricted to the beta range (13–35 Hz) with filtering using an infinite impulse response filter from 5–45 Hz of the raw data. An event-related synchronization/desynchronization was initially identified and confirmed by visual inspection of spectral plots and subsequent statistical analysis.

For statistical analysis, the mean firing rate was compared during the task and baseline under the null hypothesis that the Vim firing rate was the same as the baseline during the silent counting of deviant tones and the standard tones. In the wrist flicking task, we compared the firing rate before the task and during the task with the null hypothesis that this motor task did not change the firing frequency. For the LFPs beta band, we divided the data into four epochs according to the average beta power response. We defined a pre-task or baseline period from −0.2 to 0 s, then an “early response window” during the 200 ms tone occurrence from 0.01 to 0.2 s, then a “cognitive window” from 0.21 to 0.6 s, and finally a “recovery or rebound” period from 0.6–1 s. The comparison between different times was done using repeated measures anova. An anova was also used to test the difference in the firing rates before, during, and after the task in Vim and Voa/Vop. All hypotheses were tested through either the non-parametric Mann–Whitney U test or parametric paired t-test, two-tailed. The Mann–Whitney U test was used to compare LFPs averages between deviant and standard, and to compare LFPs averages for different diseases. All data presented were mean ± SD. *P*-values < 0.05 was considered significant evidence to reject the null hypothesis.

### Reporting summary

Further information on research design is available in the Nature Portfolio Reporting Summary linked to this article.

## Supplementary information

Supplementary figures and table

Reporting summary checklist

## Data Availability

All relevant data is available upon request pending approval of a data sharing agreement with our institute. Please contact the corresponding author for requests.

## References

[1] Broadbent DE (1957). A mechanical model for human attention and immediate memory. Psychol. Rev..

[2] Treisman AM (1964). Selective attention in man. Br. Med Bull..

[3] McLeod, S. A. *Selective attention*. *Simply Psychology*, www.simplypsychology.org/attention-models.html (2018).

[4] Noudoost B, Chang MH, Steinmetz NA, Moore T (2010). Top-down control of visual attention. Curr. Opin. Neurobiol..

[5] Crick F (1984). Function of the thalamic reticular complex: the searchlight hypothesis. Proc. Natl Acad. Sci. USA.

[6] Treisman A (1982). Perceptual grouping and attention in visual search for features and for objects. J. Exp. Psychol. Hum. Percept. Perform..

[7] Pinault D (2004). The thalamic reticular nucleus: structure, function and concept. Brain Res Brain Res Rev..

[8] McAlonan K, Cavanaugh J, Wurtz RH (2008). Guarding the gateway to cortex with attention in visual thalamus. Nature.

[9] Wimmer RD (2015). Thalamic control of sensory selection in divided attention. Nature.

[10] Kam JWY, Solbakk AK, Endestad T, Meling TR, Knight RT (2018). Lateral prefrontal cortex lesion impairs regulation of internally and externally directed attention. Neuroimage.

[11] Nakajima M, Schmitt LI, Halassa MM (2019). Prefrontal cortex regulates sensory filtering through a basal ganglia-to-thalamus pathway. Neuron.

[12] Albaugh DL, Huang C, Ye S, Pare JF, Smith Y (2021). Glutamatergic inputs to GABAergic interneurons in the motor thalamus of control and parkinsonian monkeys. Eur. J. Neurosci..

[13] Rauschecker JP (1998). Cortical control of the thalamus: top-down processing and plasticity. Nat. Neurosci..

[14] Zikopoulos B, Barbas H (2006). Prefrontal projections to the thalamic reticular nucleus form a unique circuit for attentional mechanisms. J. Neurosci..

[15] Lenz FA (1990). Single unit analysis of the human ventral thalamic nuclear group. Activity correlated with movement. Brain.

[16] Ohye C (1989). Further physiological observations on the ventralis intermedius neurons in the human thalamus. J. Neurophysiol..

[17] Rouiller EM, Liang F, Babalian A, Moret V, Wiesendanger M (1994). Cerebellothalamocortical and pallidothalamocortical projections to the primary and supplementary motor cortical areas: a multiple tracing study in macaque monkeys. J. Comp. Neurol..

[18] Rohani M, Fasano A (2017). Focused ultrasound for essential tremor: review of the evidence and discussion of current hurdles. Tremor Other Hyperkinet Mov..

[19] Bockova M (2011). Involvement of the subthalamic nucleus and globus pallidus internus in attention. J. Neural Transm..

[20] Alanazi FI (2021). Neurophysiological responses of globus pallidus internus during the auditory oddball task in Parkinson’s disease. Neurobiol. Dis..

[21] Klostermann F (2006). Mental chronometry of target detection: human thalamus leads cortex. Brain.

[22] Basha D (2014). Beta oscillatory neurons in the motor thalamus of movement disorder and pain patients. Exp. Neurol..

[23] McCusker MC, Wiesman AI, Schantell MD, Eastman JA, Wilson TW (2020). Multi-spectral oscillatory dynamics serving directed and divided attention. Neuroimage.

[24] Spitzer B, Haegens S (2017). Beyond the Status Quo: A Role for Beta Oscillations in Endogenous Content (Re)Activation. eNeuro.

[25] Chang A, Bosnyak DJ, Trainor LJ (2018). Beta oscillatory power modulation reflects the predictability of pitch change. Cortex.

[26] Te Woerd ES, Oostenveld R, de Lange FP, Praamstra P (2017). Impaired auditory-to-motor entrainment in Parkinson’s disease. J. Neurophysiol..

[27] Wehrman J, Sowman P (2021). Oddball onset timing: Little evidence of early gating of oddball stimuli from tapping, reacting, and producing. Atten. Percept. Psychophys..

[28] Kuhn AA (2008). High-frequency stimulation of the subthalamic nucleus suppresses oscillatory beta activity in patients with Parkinson’s disease in parallel with improvement in motor performance. J. Neurosci..

[29] Kulashekhar S, Pekkola J, Palva JM, Palva S (2016). The role of cortical beta oscillations in time estimation. Hum. Brain Mapp..

[30] Hutchison WD (2004). Neuronal oscillations in the basal ganglia and movement disorders: evidence from whole animal and human recordings. J. Neurosci..

[31] van Schouwenburg M, Aarts E, Cools R (2010). Dopaminergic modulation of cognitive control: distinct roles for the prefrontal cortex and the basal ganglia. Curr. Pharm. Des..

[32] Pilgrim MJD, Ou ZA, Sharp M (2021). Exploring reward-related attention selectivity deficits in Parkinson’s disease. Sci. Rep..

[33] Kurata K (2005). Activity properties and location of neurons in the motor thalamus that project to the cortical motor areas in monkeys. J. Neurophysiol..

[34] Fuster JM (1973). Unit activity in prefrontal cortex during delayed-response performance: neuronal correlates of transient memory. J. Neurophysiol..

[35] MacMillan ML, Dostrovsky JO, Lozano AM, Hutchison WD (2004). Involvement of human thalamic neurons in internally and externally generated movements. J. Neurophysiol..

[36] Prochazka A (2021). Proprioception: clinical relevance and neurophysiology. Curr. Opin. Physiol..

[37] Raethjen J (2008). Provocation of Parkinsonian tremor. Mov. Disord..

[38] Wilken M, Rossi M, Rivero AD, Hallett M, Merello M (2019). Re-emergent tremor provocation. Parkinsonism Relat. Disord..

[39] Li Y (2020). Aberrant advanced cognitive and attention-related brain networks in Parkinson’s disease with freezing of gait. Neural Plast..

[40] Peterson DS, King LA, Cohen RG, Horak FB (2016). Cognitive contributions to freezing of gait in Parkinson disease: implications for physical rehabilitation. Phys. Ther..

[41] Lei J (2019). Cognitive effects of rhythmic auditory stimulation in Parkinson’s disease: a P300 study. Brain Res..

[42] Sharma VD, Patel M, Miocinovic S (2020). Surgical treatment of Parkinson’s disease: devices and lesion approaches. Neurotherapeutics.

[43] Li Y (2020). Distinct subnetworks of the thalamic reticular nucleus. Nature.

[44] Wells MF, Wimmer RD, Schmitt LI, Feng G, Halassa MM (2016). Thalamic reticular impairment underlies attention deficit in Ptchd1(Y/-) mice. Nature.

[45] Trenado C, Haab L, Strauss DJ (2009). Corticothalamic feedback dynamics for neural correlates of auditory selective attention. IEEE Trans. Neural Syst. Rehabil. Eng..

[46] Lenz FA (1988). Single unit analysis of the human ventral thalamic nuclear group: correlation of thalamic “tremor cells” with the 3-6 Hz component of parkinsonian tremor. J. Neurosci..

[47] Schaltenbrand, G., Wahren, W., Hassler, R. G. *Atlas for stereotaxy of the human brain* (Thieme, 1977).

[48] Fawcett AP, Dostrovsky JO, Lozano AM, Hutchison WD (2005). Eye movement-related responses of neurons in human subthalamic nucleus. Exp. Brain Res..

