# Supplementary material for: Top-down control of human motor thalamic neuronal activity during the auditory oddball task
Source: NPJ Parkinsons Dis. 2023 Mar 27;9:46. doi: 10.1038/s41531-023-00493-1 (PMC10042852; doi:10.1038/s41531-023-00493-1)
Supplement: Supplementary file 1 — Supplementary figures and table [file 41531_2023_493_MOESM1_ESM.pdf]

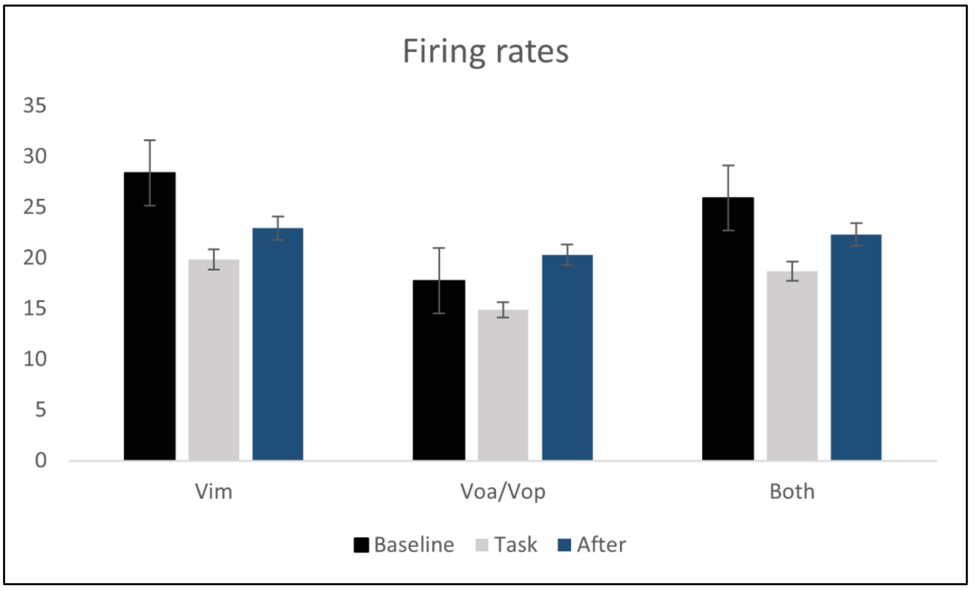

Supplementary figure 1. The firing rates of motor thalamic neurons. the bars represent firing rate across trials before, during, and after the auditory oddball task in the two regions (Vim, Voa/Vop). There is a clear decrease in the firing rates during the task and a rebound after the task. This decrease was statistically significant in anova. Results are mean ± SEM.

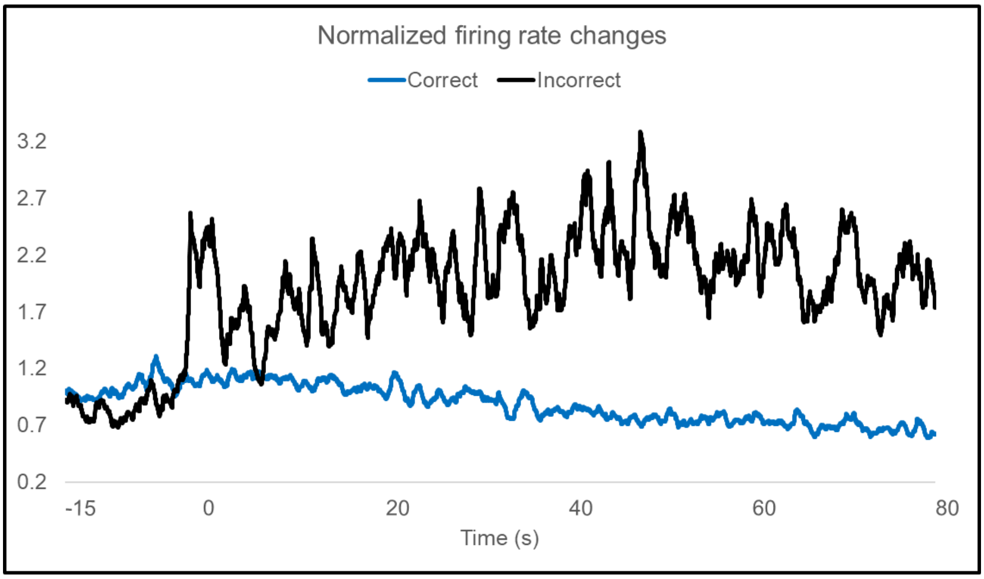

Supplementary figure 2. Motor thalamic neurons firing rate in tasks that counted correctly and incorrectly. Normalized Vim and Voa/Vop neuronal firing rate in correct (Blue) and incorrect (black) groups. The correct number of deviant tones was reported in 19 patients (76%) while six patients (24%) reported an incorrect number of tones. Tasks were classified as correct if the count was ±1 to the actual number of deviant tones. Zero is the beginning of the task.

	DEVIANT			STANDARD		
**Region**	INC	DEC	NR	INC	DEC	NR
**Vim (38)**	14 (36.8%)	16 (42.1%)	8 (21.1%)	6 (15.8%)	3 (7.9%)	29 (76.3%)
**Vop/Voa (18)**	3(16.7%)	11(61.1%)	4(22.2%)	4 (22.2%)	0	14 (77.8%)

Supplementary table 1. The distribution of different response observed in Vim and Voa/Vop. This table is showing the frequency of different responses with their distribution in the motor thalamus. A total of 56 cells and Most of these neurons in both the Vim and Voa/Vop showed a response (either increase or decrease) in the firing rate to the deviant tones but not to the standard tones.
