# Supplementary material for: Top-down control of human motor thalamic neuronal activity during the auditory oddball task
Source: NPJ Parkinsons Dis. 2023 Mar 27;9:46. doi: 10.1038/s41531-023-00493-1 (PMC10042852; doi:10.1038/s41531-023-00493-1)
Supplement: Supplementary file 2 — Reporting summary checklist [file 41531_2023_493_MOESM2_ESM.pdf]

nature portfolio

Corresponding author(s): *Double-blind peer review submissions: write DBPR and your manuscript number here instead of author names.*

Last updated by author(s): *YYYY-MM-DD*

Reporting Summary

Nature Portfolio wishes to improve the reproducibility of the work that we publish. This form provides structure for consistency and transparency in reporting. For further information on Nature Portfolio policies, see our Editorial Policies and the Editorial Policy Checklist.

Statistics

For all statistical analyses, confirm that the following items are present in the figure legend, table legend, main text, or Methods section.

n/a	Confirmed	
☐	☒	The exact sample size (*n*) for each experimental group/condition, given as a discrete number and unit of measurement
☒	☐	A statement on whether measurements were taken from distinct samples or whether the same sample was measured repeatedly
☐	☒	The statistical test(s) used AND whether they are one- or two-sided<br>*Only common tests should be described solely by name; describe more complex techniques in the Methods section.*
☒	☐	A description of all covariates tested
☒	☐	A description of any assumptions or corrections, such as tests of normality and adjustment for multiple comparisons
☐	☒	A full description of the statistical parameters including central tendency (e.g. means) or other basic estimates (e.g. regression coefficient) AND variation (e.g. standard deviation) or associated estimates of uncertainty (e.g. confidence intervals)
☐	☒	For null hypothesis testing, the test statistic (e.g. *F*, *t*, *r*) with confidence intervals, effect sizes, degrees of freedom and *P* value noted<br>*Give P values as exact values whenever suitable.*
☒	☐	For Bayesian analysis, information on the choice of priors and Markov chain Monte Carlo settings
☒	☐	For hierarchical and complex designs, identification of the appropriate level for tests and full reporting of outcomes
☒	☐	Estimates of effect sizes (e.g. Cohen's *d*, Pearson's *r*), indicating how they were calculated

Our web collection on statistics for biologists contains articles on many of the points above.

Software and code

Policy information about availability of computer code

Data collection	*Provide a description of all commercial, open source and custom code used to collect the data in this study, specifying the version used OR state that no software was used.*
Data analysis	*Provide a description of all commercial, open source and custom code used to analyse the data in this study, specifying the version used OR state that no software was used.*

For manuscripts utilizing custom algorithms or software that are central to the research but not yet described in published literature, software must be made available to editors and reviewers. We strongly encourage code deposition in a community repository (e.g. GitHub). See the Nature Portfolio guidelines for submitting code & software for further information.

Data

Policy information about availability of data

All manuscripts must include a data availability statement. This statement should provide the following information, where applicable:

- Accession codes, unique identifiers, or web links for publicly available datasets
- A description of any restrictions on data availability
- For clinical datasets or third party data, please ensure that the statement adheres to our policy

Due to patient confidentiality regulations at our institute, data is not open access. However, upon contact with the authors and reasonable requests, a data sharing agreement with our institute may be entered into. Please contact WDH for requests.

Human research participants

Policy information about studies involving human research participants and Sex and Gender in Research.

Reporting on sex and gender	Parkinsons disease and essential tremor affect both sexes and patient's are offered surgery based on the severity of tremor and its impact activities of daily living and quality of life. There are more men than women that opt for DBS so our sample sex ratio is affected by this but oddball performance has no basis to suspect sex differences and the small sample size may decrease the power of such a comparison. No gender data is collected in the movement disorders clinic to my knowledge.
Population characteristics	The study involved a consecutive series of medically refractory movement disorders patients who were selected by the neurosurgical, neurology and psychology team to undergo deep brain stimulation surgery for the treatment of tremor. Patients were off medications for 12 hours and have intact cognitive function to be able to complete the task.
Recruitment	There was no selection bias for patients recruitment, since a consecutive series of consenting patients was studied. Patients met standard criteria for DBS surgery for tremor and gave free and informed consent before asked to do the task.
Ethics oversight	The study conformed to the guidelines set by the Tri-Council Policy on Ethical Conduct for Research Involving Humans and were approved by the University Health Network Research

Note that full information on the approval of the study protocol must also be provided in the manuscript.

Field-specific reporting

Please select the one below that is the best fit for your research. If you are not sure, read the appropriate sections before making your selection.

☒ Life sciences ☐ Behavioural & social sciences ☐ Ecological, evolutionary & environmental sciences

For a reference copy of the document with all sections, see nature.com/documents/nr-reporting-summary-flat.pdf

Life sciences study design

All studies must disclose on these points even when the disclosure is negative.

Sample size	The sample size was defined using power analysis from previous studies . The study involved patients with disabling tremor and undergo DBS surgery.
Data exclusions	Some neurophysiology data was excluded either due to noise involvement or technical difficulties.
Replication	Although we described various types of neuronal responses, these are based on action potential firing which is a fundamental neurophysiological characteristic of the motor thalamus and our neuronal responses replicate those found in non human primate species. In addition, the selective response to deviant tones in the oddball task is a robust finding across both our previous studies in globus pallidus as well as in many other neurophysiological and neuroimaging studies.
Randomization	This study is a neurophsyiolgical research study and not a clinical trial, so some of these categories do not apply. There was no active drug arm or other group comparison made.<br>Since a sequential series of patients were studies this was random to the extent that whoever had completed preoperative evaluation and work up was scheduled for surgery.
Blinding	N/A

Reporting for specific materials, systems and methods

We require information from authors about some types of materials, experimental systems and methods used in many studies. Here, indicate whether each material, system or method listed is relevant to your study. If you are not sure if a list item applies to your research, read the appropriate section before selecting a response.

Materials & experimental systems

n/a	Involved in the study
☒	☐ Antibodies
☒	☐ Eukaryotic cell lines
☒	☐ Palaeontology and archaeology
☒	☐ Animals and other organisms
☒	☐ Clinical data
☒	☐ Dual use research of concern

Methods

n/a	Involved in the study
☒	☐ ChIP-seq
☒	☐ Flow cytometry
☒	☐ MRI-based neuroimaging
